# Telepointer technology in telemedicine : a review

**DOI:** 10.1186/1475-925X-12-21

**Published:** 2013-03-09

**Authors:** Rohana Abdul Karim, Nor Farizan Zakaria, Mohd Asyraf Zulkifley, Mohd Marzuki Mustafa, Ismail Sagap, Nani Harlina Md Latar

**Affiliations:** 1 Department of Electrical, Electronic & Systems Engineering, Faculty of Engineering & Built Environment, Universiti Kebangsaan Malaysia (UKM), Bangi, Malaysia; 2 Faculty of Electrical & Electronics Engineering, Universiti Malaysia Pahang, Pekan, Malaysia; 3 Department of Surgery, Universiti Kebangsaan Malaysia (UKM) Medical Centre, Kuala Lumpur, Malaysia

**Keywords:** Telepointer, Telemedicine, Cursor pointer, Hand pointer, Laser pointer, Sketching pointer

## Abstract

Telepointer is a powerful tool in the telemedicine system that enhances the effectiveness of long-distance communication. Telepointer has been tested in telemedicine, and has potential to a big influence in improving quality of health care, especially in the rural area. A telepointer system works by sending additional information in the form of gesture that can convey more accurate instruction or information. It leads to more effective communication, precise diagnosis, and better decision by means of discussion and consultation between the expert and the junior clinicians. However, there is no review paper yet on the state of the art of the telepointer in telemedicine. This paper is intended to give the readers an overview of recent advancement of telepointer technology as a support tool in telemedicine. There are four most popular modes of telepointer system, namely cursor, hand, laser and sketching pointer. The result shows that telepointer technology has a huge potential for wider acceptance in real life applications, there are needs for more improvement in the real time positioning accuracy. More results from actual test (real patient) need to be reported. We believe that by addressing these two issues, telepointer technology will be embraced widely by researchers and practitioners.

## Review

### Introduction

Fast and accurate long-distance communication have been very fundamental factors to humankind advancement. Telemedicine is one of the areas that benefited from the recent innovation in network and communications, which proved to be crucial in saving many lives [1]. A basic system of telemedicine typically involves communication between two or more persons that are located in different places. Some examples of telemedicine-based applications are education, surgery, consultation and many more.

Without doubt, the best mode of communication is face-to-face communication due to natural presence of gesture, interaction, deictic instructions, face expression and voice intonation, which helps in explaining the real meaning of the speech. However, long-distance communication lacks of natural presence contrary to face-to-face communication, which usually leads to misunderstand and misinterpretation of the information, especially when dealing with deictic gestures. To improve the communication quality, telepointer technology has been widely used to help the speaker in conveying the real meaning of the words. It is used as the support tool which is capable of boost up the power of distance communication through a sense of presence.

Telepointer technology can be defined as “an interaction style for presentation system interactive television, and other systems, where the user is positioned at a remote site from the display” [2]. The main function of a telepointer is to point at the specific display so that its motion could represent the human gesture. Meanwhile, display devices allow the collaborator to view the same scene as seen by the other parties. Greenberg *et al.*[3] stated that “telepointers are a natural focus of attention for group participants, and they can be leveraged to show information vital for smooth collaboration: interaction modes, system state, identity, actions of others, and so on.“ In other words, telepointer can be used 1) to point to an object or region of interest and 2) to create a pattern to signify something depending on movement, location and temporal information at the remote display.

Nowadays, a lot of applications use a camera-based physical gesture to improve the communication quality, especially hand gestures that can be used for pointing, overlaying hands and sketching [4]. Telepointer can be broadly classified into two categories; low level and high level. Low level system refers to a pointer which provides the coordinate information only such as cursor and laser pointer. Meanwhile, high level system provides more than just coordinate information by providing instruction and complicated data such as hand gestures, sketching, drawing and overlaying hands.

A variety of technologies have been developed to facilitate telepointer gesturing, such as GestureMan [5], DOVE-Drawing over Video Environment [6], Mixed Ecology [7] and Head mounted display (HMD) [8]. GestureMan employs a robotic system that represents the gesture through a laser pointer. DOVE system creates the sketching gestures by overlaying pen-based gestures on a tablet personal computer (PC) and Mixed Ecology employs unmediated gestures. HMD based on augmented reality by superimposed pointer on a video channel.

### Basic system telepointer

**Figure 1:**
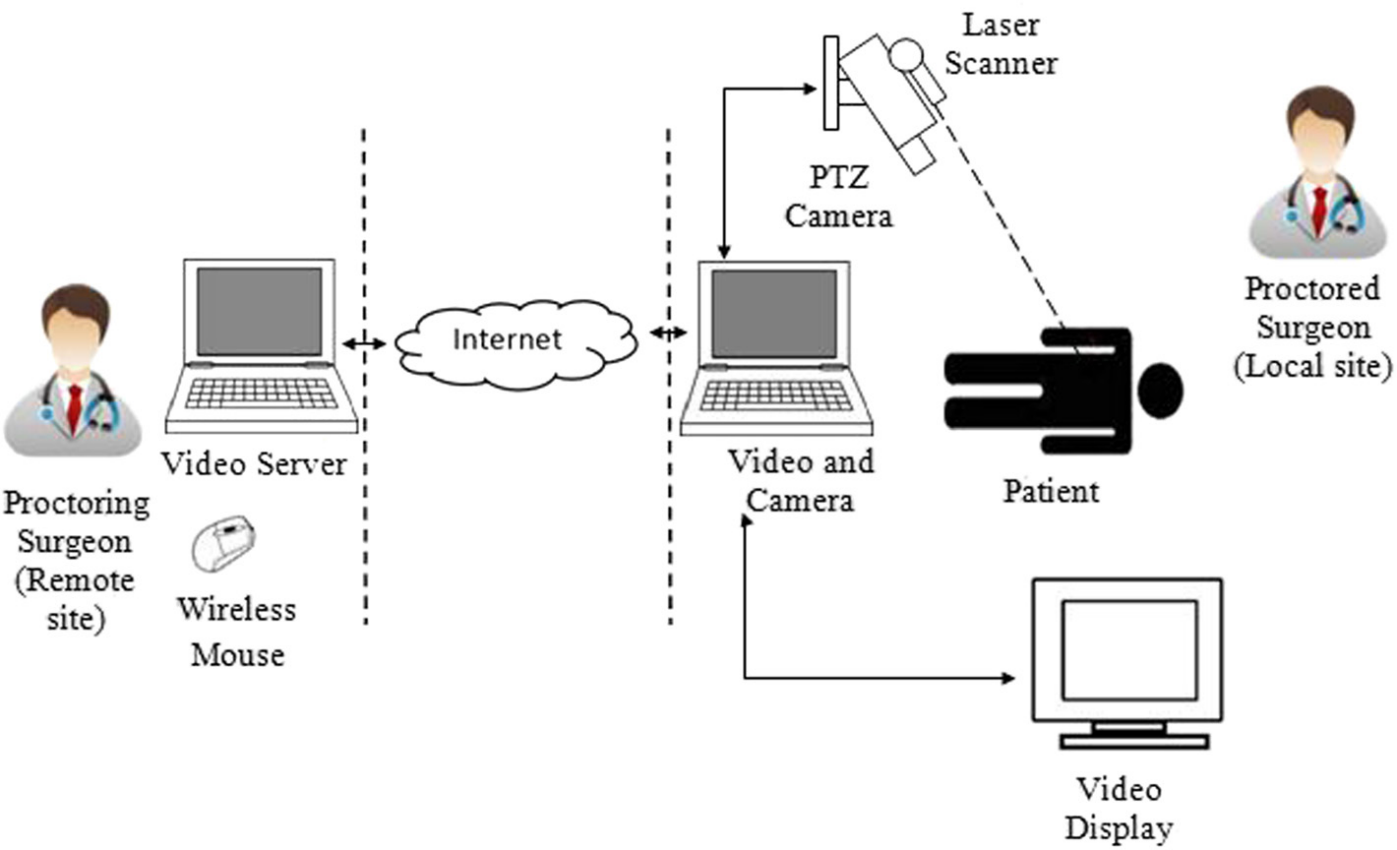
**Typical telepointer system applied in telemedicine and laser as a pointing device (adapted from Ereso*, et al. ***[9]**).**

Telepointer is vital elements in remote collaboration and computer supported co-operative work (CSCW). CSCW is a combination of hardware and software resources to allow groups to collaborate either in static or mobile environments. Static refer to fix confined workspace while mobile refer to moving workspace either remote or local user or both. Hence, technical setup and hardware system almost inherit from both areas. A simple mechanism is used to set up a telepointer system which consists of a video display; a pointer device; a computer; and a camera as a means of communication with the collaborator. A camera is used to capture the visual information at the local site which is then transmitted to the remote site. The local site will have a pointing device (e.g.: cursor mouse) to point to a particular object on the computer display which should be synchronous between the local and remote sites. As a result, the other parties will be able to view the exact scene as viewed by the sender in order to reduce false information. Figure 1 shows the typical telepointer system applied in telemedicine.

### Motivation

A telepointer system offers many advantages such as 1) to provide coordinate information of the target object, 2) create the sense of presence and 3) increase the audience attention by using the multiple form of the telepointer such as colour, size and image [10]. It is difficult to rely on verbal instruction alone while giving the instruction through the internet. By adding the pointer information, the collaborator will be able to understand the command easily. Previous researches [11-14] have demonstrated that a telepointer system has been well accepted as the support tool for sending gesture information and enhance the performance of the collaborator. In addition, it also reduces the travel cost since both collaborators are not required to be at the same place for undergo their activities.

Sharing and exchanging knowledge about the treatment and diagnosis of a disease is a common practice in the medical world. It is crucial in the medical field to have effective communication mode such as discussion and consultation in order to come out with the best treatment. Lack of expert and specialist in rural areas is of great concern for the government, especially when diagnosing a rare disease and difficult symptoms. Therefore, telemedicine can be used to overcome this shortcoming. The problem becomes more complicated for an online-based surgery where a specialist needs to assist a general surgeon in a remote site who may lack the required skills. An example of successful telesurgery was given by Brévart *et al*. [15] where an emergency surgery needs to be performed to a two-year-old boy, who had a severe vertex epidural hematoma (VEDH).

As reported by McLauchlan *et al.*[16], only 32% of the junior doctors managed to successfully diagnose a trauma case based on X-ray’s data, while 80% of the senior doctors correctly diagnosed the same disease. This issue can be attributed to low confidence among the junior doctors where a second opinion is needed to help them in doing the diagnose [17-19]. Hence; a telemedicine can be employed as a support tool for facilitating the knowledge sharing. A lot of researches [20,21] believe that the telepointer technology has a great potential in the medical field, since it 1) improves the performance of doing a task, 2) increase the accuracy of the diagnose and 3) avoid the tragedy during the golden rescue minutes due to lack of specialist. According to Sachpazidis *et al.*[22], the telepointer is an important feature in collaborative applications, especially in medical collaboration. It helps a lot during long range diagnosis, consultation and mentoring by enhance the medical services and computer-mediated instructions.

However, there are two main limitations to the telepointer. Firstly, network condition will heavily affect the quality of transmit signal. Several studies [23-25] show that a major contributor to error of pointing is due to delay, which result in jitter and latency. A delay to the pointer system may cause a fatal consequence, especially in tele-surgery where a wrong location may be pointed. Secondly, current telepointer systems lack of tracking capability. Tracking allows the system to be updated by using prediction data in case of short-period signal loss.

The paper is organized into 5 sections. A brief description of Telemedicine is given in Section 2. Section 3 outlines the telepointer technologies in telemmedicine. Issues and challenges in section 4. Conclusion and future works are summarized in section 5 respectively.

### A brief description of telemedicine

**Figure 2:**
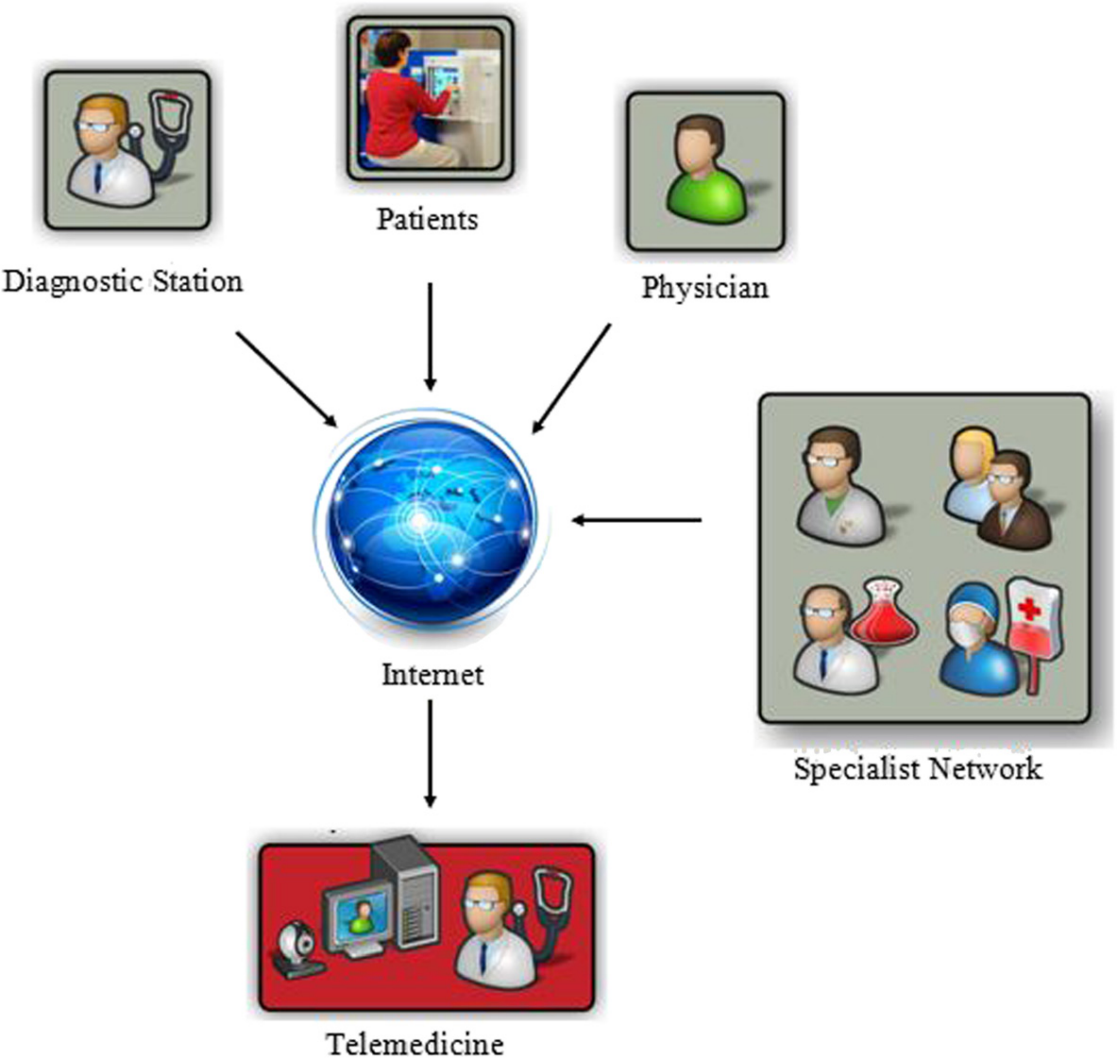
**Participants of telemedicine communicate at distance thru through internet network (adapted from **[27]**).**

Initially, telemedicine was inspired by evolution of the space technology which happened around 1960s. The system was first used to monitor astronauts’ physiological parameters by using real time wireless approach during the outer space expedition. Presently, clinical medicine that allows patient medical information to be shared through interactive multimedia has been the driving force of telemedicine application development. Rizou *et al*. [26] defines a telemedicine system as “Telemedicine is the use of electronic information and communication technologies to provide and support health care when distance separates the participants (physicians, providers, specialists and patients)” as illustrate at Figure 2. Thus, we can infer from the definition that a telemedicine system has a very limited capability to interact physically with all the involved parties except through a telecommunication mean.

Telemedicine can be broadly divided into two types of communication mode; online and offline. Offline method is the simplest approach, which requires less sophisticated equipments and technical facilities. Basically, the system just records the patient data first and then transferred to the interested party. For a teleconsultation system, medical data such as images, sound, and text are collected and stored, which is then forwarded to the medical specialist. The specialist will then diagnose the data at any convenient time and of course within the allowable time frame. Commonly, offline method was only applicable for non-critical diseases such as small skin issue and minor pathology problem. Besides, it is used to obtain a second opinion to strengthen the first doctor deduction. Several examples of popular offline communication mediums are email [28], iphone [29], Multimedia Messaging Service (MMS) [30], and Short Message Service (SMS) [18] which do not require a telepointer system in place.

Contrary to the offline method; online approach involves two ways of communication simultaneously by sending and receiving the feedback instantaneously. It provides more convenient and satisfaction [31] due to additional sense of presence. It is normally employed for the critical cases (heart disease, diabetes mellitus, cardiac), which requires face to face communication. The usual modes of online communication are video conferencing technology that integrates both multimedia elements of video and audio [32,33], desktop to desktop [34]. The setup requirement of online method is more extensive because of the extra technical equipments required such as video camera, video conferencing equipment, telepointer, computer and high-speed network.

Telemedicine has also been applied to both clinical and nonclinical applications. Clinical term refers to the task that requires direct diagnosis and treatment of the patients such as general healthcare delivery (nursing, follow up, trauma, rehabilitation, pharmacy) and specialist care delivery (cardiology, pathology, dermatology, surgery). On the other hand, nonclinical is any task that does not involve treatment and diagnosis of the patient yet still related to the patient care such as distance learning for sharing knowledge and information, pre-operation meeting, medical appointment and many more. Both clinical and nonclinical job can be enhanced by implementing a telepointer system for more efficient communication.

There are several subbranches under the telemedicine system such as teleconsultation, telementoring, teleproctoring, telesurgery, telediagnose, telepresence, teletrauma and many more. All of these branches can be perceived as a cheaper alternative to the traditional methods [35] except for the telesurgery. Telesurgery is the most sought over service and requires the most advance facilities to operate on. The surgeons will remotely control the robot action that located in the operation theatre by using a computer interface. One of the earliest successful telesurgery was achieved by operation Lindbergh in 2001 [36]. It was the first surgery where the operating surgeon was removed from the operating room to perform minimally invasive surgery on a human patient. The surgeon was in New York City while the patient was in Strasbourg, France. The setup cost was very expensive, which can go up to $100,000 per terminal [34]. However, most hospitals or clinics that need the system most are located in rural area of poor and developing countries, where majority of them cannot afford these terminals due to limited health care budget [37]. Besides, the system is still in early development phase, which can be very risky because of the 1) failure possibility due to clinician lack of skills, 2) technical error of the communication system and 3) quality inconsistency across geographic or economic boundaries. Hence, a good network and communication technology is a vital component for a successful telesurgery system.

As we stated before, a good communication system is very critical for an accurate telemedicine system since it will act as the medium for delivering and exchanging information from one place to another place. Performance of a telemedicine system always dependent on the network bandwidth which gives the upper limit of information transferred. In order to receive instantaneously accurate information, a telemedicine system required a high bandwidth network to transmit complex medical information in a real-time. The main downside of such a system is a higher installation and maintenance cost. For example, Nagata and Mizushima [34] has shown that a medical image in Joint Photographic Experts Group (JPEG) format of 1000 x 1000 pixels requires 10 to 65 seconds to be uploaded to four different clients, while an image of 640 x 480 pixels had a much faster response time (2-5 s) with 1.5Mbit/s connection. Besides, choice of the equipments, transmission media and network bandwidth are dependent on the remote hospital location. As for example, a telemedicine system in Madagascar cannot operate on a high bandwidth system due to limited technologies [38]. They can only manage a system with low cost equipments for 25 kbit/s bandwidth connection. Therefore, only small-sized data such as electrocardiogram (ECG) and blood pressure can be transmitted for a real time teleconsultation.

### Telepointer technology in telemedicine

**Figure 3:**
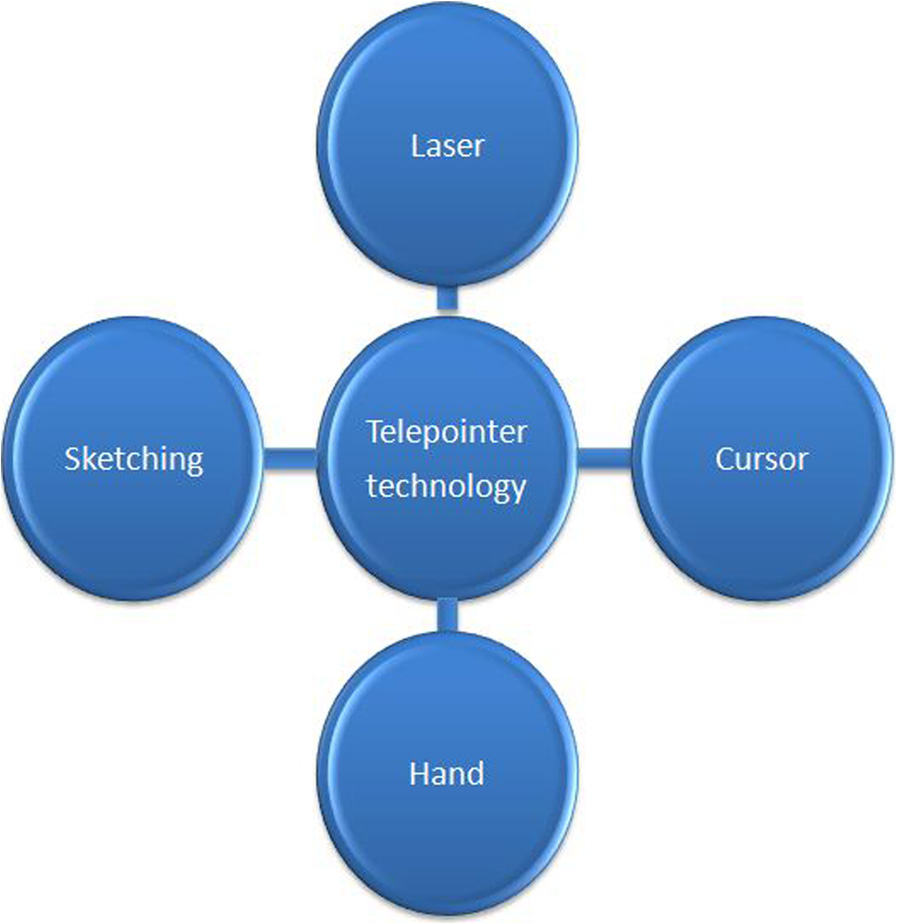
Division of the telepointer technology used in telemedicine.

Current technology in telemedicine allows the patient image to be transmitted in real time to the expert by using a camera. A two-way communication is established via an audio-visual tool, which enables the general clinicians and the expert to view the same video as displayed simultaneously on the expert’s terminal. There are several approaches in telepointer where the most popular methods utilize a laser pointer, cursor movement or sketching to generate a mutually visible remote pointer in the shared workspace. Figure 3 shows the division of the telepointer technology used in telemedicine, which will be discussed in the following subsections.

### Cursor pointer

Cursor pointer can be regarded as the simplest pointer. It is just a small graphical pointer on the screen display, which normally takes an arrow form. Location and movement of the cursor are controlled by an external computer device such as mouse or touchpad. Numerous studies have explored the usage of the cursor pointer for the remote collaboration [24,39,40] and have been proven to be very important in enhancing the performance of the collaborative tasks. Kirk and Fraser [7] found that the performance of overlaying hand approach was better than the actual sketch devices (for example, cursor pointer, pen) in terms of the effectiveness. However, sketch device has a superior performance compared to a simple laser pointer.

**Figure 4:**
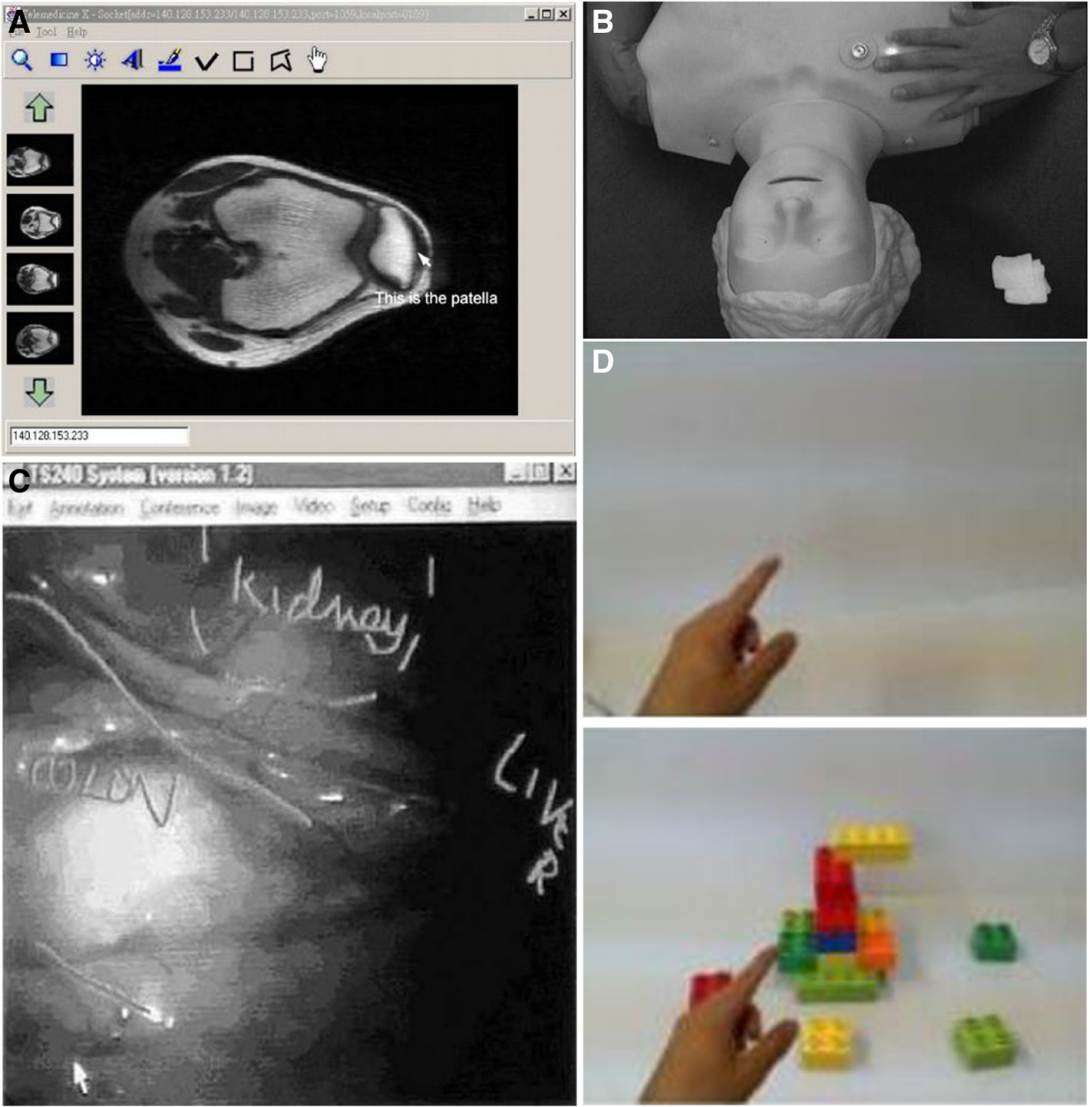
**(A) cursor pointer applied in teleradiology (adapted from **[43]**) (B) laser pointer applied in telementoring (adapted from **[45]**) (C) telestrator applied in laparoscopic (adapted from **[46]**) (D) hand pointer (adapted from **[21]**.**

In telemedicine, cursor pointer plays the main role in showing and stressing the details of the examined image. It can be used to highlight any specific location and distribution of a lesion which has been applied in the majority of the teleconsultation systems. In [41], Julsrud *et al.* explored the capability of a cursor pointer system for congenital heart disease consultation. They have performed an initial test of 54 sessions of teleconsultation comprised of 38 patients with various types of congenital heart disease. The results were promising since 72 (67%) respondents out of 108 observations believed that a cursor pointer was helpful during the consultation. Hence, the study suggested that the implementation of a cursor pointer will enhance the consultation process, especially for congenital heart disease. Moreover, the system has also been implemented in teleradiology consultation [42,43] as shown in Figure 4(A). A similar approach has been adopted in telementoring of gynecological surgery where a cursor pointer is used to highlight the structure and landmark anatomy on the monitor of the operating room [44]. For this case, the cursor movements have been synchronized between the local and the remote sites.

Generally, a cursor pointer system in telemedicine can be classified into two modes of communication scheme; 1) master and slave and 2) groupware. Both schemes apply the concept of “What you see is what I see”. It means that each user could see what the other parties were pointing. Master and slave scheme involves two parties where the collaboration only occurs if one person situated at the local site while the other person at the remote site. This scheme was designed to prevent any competition while operating the telepointer such as the cursor and laser pointer [22,47]. Hence, only one person will be granted the authority to control the cursor pointer at one time. The slave site can become the master just by pressing a special button to reverse the authority. Groupware scheme involves more than two parties where each user can see their own action and other users’ cursor pointer. Anyone can control the other parties’ pointers. In the work by Lee *et al.*[43], they faced inconsistency issue with the cursor pointer when too many users activate the system simultaneously. This issue can be solved by using a simple solution such as token passing scheme.

Some studies have focused on improving the attention and gaze awareness among the collaborators by enhancing the visual properties of the cursor. By default, a cursor pointer is usually small and sometimes its movement may not be noticeable by others. It will be worse if the display is situated far away from the user. Alternatively, Sachpazidis *et al.*[22] proposed changeable graphical symbol to represent the cursor when it is in active mode. The changeable scheme has also been implemented in the hand cursor system. A blink of yellow flash is added below the cursor to indicate the authority transfer to the remote site. If the transfer is successful, the cursor colour will be changed to grey. Usually, a cursor pointer has an embedded capability to zoom in and out just by clicking the right and left side of the mouse. In the system developed by Nagata and Mizushima [34], the cursor shape was changed to an arrow, and the user can define their own cursor colour. For a groupware application, cursor pointers of each participant are marked by their name and host address of the remote computers [48].

Real time communication software have been developed to improve high-level telemedicine system. Some examples of existing methods are; 1) wavelet-based interactive video communication system (WinVicos) [49] 2) TeleDicom [50] 3) REmote Patient Education in a Telemedicine Environment Architecture (REPETE) [51]. All of these methods used sophisticated communication software, which consists of all minimum requirements for a collaboration scheme such as audio, interactive video conference system, still images and a telepointer. All of these softwares used cursor as a pointer for deictic referencing and gesturing. However, Huang *et al.*[21] claimed that a simple cursor pointer was insufficient for an effective collaboration.

### Laser pointer

The first laser pointer was invented in 1960 [52] with the intention of replacing the traditional pointer such as a hand-held wooden stick for more flexible presentation. The main advantages of the device are long range pointer capability and ability to function well in a low ambient surroundings. It produces a bright spot of light to attract audience attention. It can be broadly classified according to its power consumption, either low or high. A low power pointer usually requires 1mWto 500 mW while a high power device can consume power from 1000 mW up to 3000 mW.

Laser pointers have three primary colours; red, green and blue. Each colour have a different range of wavelength with red laser has the highest wavelength, followed by green and blue. Red and green colours are most suitable to be implemented in a task that requires the attention from the audience [53]. Unfortunately, red laser have similar colour to blood and human tissue, which makes it not suitable for pointing in clinical applications. Hence, the usual practice is to implement green laser, which have a good contrast to blood and tissue colour. Paper by Schneider*, et al.*[54] claimed that green laser performed well enough in the operative field for clinical remote consultation.

During its early development, laser pointer system is usually coupled with the monitor interface. It just not acting as a pointer, but able to navigate the display as well as function as a mouse [55-57]. The advantages telepointer usage in telemedicine are 1) it can directly point to the interest object, 2) it requires a very low bandwidth, 3) it is not an invasive procedure, 4) it can be easily incorporated into the modern operating theatre and 5) low cost installation.

Nowadays, laser technology is used widely in telemedicine not just for pointing purpose since a high power device have been used in surgical operation to cut through tissue, seal and cauterize wounds while a low power device can be used to treat injury and speed-up the healing process. Usually, for a pointer application, a diode laser pointer will have a power less than 1 mW power [45]. It has been used to improve teaching efficiency during surgical education of a laparoscopic surgery. Ursic *et al.*[58] used a common pen-sized laser pointer to mark on the video screen to support and accelerate interaction between the surgeons and their assistants. Laser pointer can also pinpoint the correct entry points as demonstrated by Racz and Kao [59]. Moreover, it is used to guide the trainees while learning the anaesthesia procedures. The surgeon also benefitted from the system since a laser pointer can act as the user input to a robotic system before they perform the medical procedure autonomously [60]. Similarly, laser pointers have also been applied in Computer Assisted Orthopaedic Surgery for locating landmarks intra-operatively [61]. Figure 4(B) shows laser pointer marks on patient body which applied in telementoring.

Preliminary research for the remote laser pointer was started by Yamazaki *et al.*[62] when they realized a fixed computer does not support the interaction between space and the real objects. Output of this research has allowed them to come out with a new idea specially for remote medical instruction. They developed a system that allows the remote expert to control laser pointer movement by using a mouse cursor, which acts as a pointer that can be directed to any particular area of interest on the patient’s body [45]. By using cameras, the system enabled both local and remote sites to share the same visual data. A similar approach was employed by Ko and Razvi [63] and Schneider *et al.*[54], in which a laser pointer was integrated to a camera that will point out the relevant anatomical points of interest as directed remotely by an expert. In [9], Ereso*, et al.* developed a system that allowed the camera to track the hand of the local surgeon. However, none of the previous publications provide much detail on the image processing part of the laser pointer.

There are other technologies that are able to support two ways communication directly such as tabletop [64], Wearable Active Camera/Laser (WACL) [8], and HandsOnVideo [65]. However, most of these systems require a complex technical setup and can only function well under limited environments for the gesture and interaction to be recognizable. Moreover, it is a difficult task for the surgeon to maintain the stability of the camera during a surgery, which might distract their concentration. Besides, it is difficult to make an accurate diagnosis and observation on the remote site when the captured image is blurred due to unstable camera effect. Furthermore, laser pointer movement depends only on the cursor movement by the expert which might lead to an error, especially while clicking the interface or taking a glance at the patient’s body. This error will lead to the inaccurate area of interest.

Results in [9,45] have shown that the presence of a laser pointer device can increase the performance speed of the surgeons. As an example, such a device can enable inexperienced surgeons to identify correctly the intended location of the surgery and enhance their chance of doing the job right on the first trial. Without the support by a laser pointer system, the novice surgeons took a longer time to complete the task and even some of them need a second trial.

### Sketching

Sketching is a basic skill in painting and drawing. In a telepointer system, sketching can be regarded as a gesture system with the added ability of pointing. The user is given the freedom to draw anything without any restriction such as any shape, line and even writing a word. Normal implementation of a sketch pointer is by sharing the drawing activities or “virtual sketchbook” among the collaborators [66]. Video input is used to provide a common drawing surface where each collaborator can view the combined image of all the sketches made by the collaborators. The work by Ou *et al.*[6] proposed a method to integrate visual information and gesture beyond the static surface like a sketchbook. They implemented a system of dynamic surface in the collaborative physical task environment. Their system can support pointing and gesture activity by using the sketch pointer on a video stream. Stylus, touch screen and telestrator are used as a drawing tool that allows its operator to sketch an overlay over an image generated by the camera.

Telestrator has a special ability that allows the user to draw on the television screen by using a particular stylus pen. It was invented in the late 1950s and widely used in advanced application of telemedicine such as telesurgery [67], teleproctoring and telementoring [68]. Telestrator managed to improve performance of a clinician who has the fundamental knowledge about the surgery yet limited or no experience in the operation room. Ali *et al.*[69] stated that telestrator is an important teaching tool, especially for minimally invasive surgery or also known as laparoscopic. Telestrator commonly used as annotation tools for directing the surgeon by highlighting the point of interest that allows better demonstration of the anatomical structure. It can also be used to lead the surgeon where to place the instruments, other than pointing out any operative threat that he might not aware. An example of such a system is used in telementoring of an adrenalectomy laparoscopic [46] as indicate in Figure 4(C). A surgeon who is an expert in open surgery and skiilful in laparoscopic procedure but limited experienced in laparascopic adrenalectomy has been guided by another expert from a remote place to perform the laparoscopic successfully. He needs to identify and recognize all the difficult structures, such as vena cava, adrenal veins, and in spleen medialization in order to complete the operation. Here, telestrator is used to guide the surgeon to identify and recognize the difficult structures. The result showed that the operating time was shorter compared to the other authors report and at the same time no complication to the patient is reported. We strongly believed that the implementation of a telestrator enhances the learning experience and accelerates the learning curve of for the laparoscopic procedure.

Conventional telestrator have a limitation on the number of participants. It operates on one-to-many relationship where only the remote site is allowed to manipulate the pointer while the local site can only view the output. The system will feed the information back to the remote site through audio information. This restriction makes a telestrator system unsuitable for a groupware application that requires multiple input from all the users. Besides, communication architecture of a conventional telestrator does not allow the users to recover back the original video. As the technology evolves, new form of multimedia framework for the telestrator was developed. Qiru and Dong [70] presented an approach that provides a platform for *n*-way Interactive Visual Content Sharing and Telestration (IVCST). The architecture of the system allows multiple users at remote sites to sketch on a shared visual content. Each user is identified by using a name tag and different sketch colour. A conventional telestrator system utilizes 2-dimensional visual system which overlaps the pointer with the points of interest. This approach reduces the accuracy of the telestrator from the user point of view. To lessen this effect, Ali *et al*. [69] investigated a 3-dimensional system for potential integration with the telestrator system. Early results showed that the system is feasible for further development since it receives positive feedback from the doctors. In addition, it provides more diverse spatial information such as 3-dimensional sense of presence and can be used for depth perception, which results in more accurate pointing position.

The popularity of a stylus pointer in touch screen tablet and personal digital assistant (PDA) technology were increased due to wide spread usage in telepointer and telemedicine applications. Both technologies allow the remote user to be at more dynamic position compared to the telestrator technology which requires the remote user to be at a static place. The main advantage of a pen-based pointer compared to other telepointer modalities (cursor, laser pointer) is the user friendly factor since it imitates the way human write on a piece of paper. Kim *et al.*[71] claimed that “pen-based computing provides people with an intuitive way to use a computer” because the user can leverage their writing skill without having to learn how to operate the keyboard and mouse. Therefore, a patient with low computer skill can still be able to provide gesture feedback to the doctor during a teleconsultation session. Dante [72] examined the usability of a pen-based pointer among the novice older users. The results revealed that majorities of the participant were satisfied with the pen-based pointer compared to the cursor since they can focus their hands and eyes at the same location. Meanwhile, cursor pointer requires them to focus on two different parts; 1) a mouse for moving the cursor and 2) view the display in order to locate the cursor on the screen. Hence, it is suitable for the patient with Parkinson’s disease [73,74]. In the work by Tu *et al.*[75], they explored the feasibility of using pen and finger-based pointer for the touch screen application. They found out that both pointers have their own advantages depending on the types of applications.

### Hand pointer

Hand pointer is a subset of the hand gesture system that mainly utilizes finger as a pointer. Fussell*, et al.*[4] classified the hand gesture system into four categories; 1) deictic (pointing), 2) iconic, 3) spatial or distance and 4) kinetic motion. It is considered as a nonverbal communication method since it is portrayed by human action and body language. There are many forms and shapes of the hand gesture that dependent heavily on the culture and norm of the society. Several examples of the human gesture technology are implemented in head tracking [76] and eye tracking [77] systems where the users are allowed to interact directly with the computer without using the mouse.

Hand pointer system is an active research area, especially for remote collaboration on a physical task [4,7,13,78]. Basically, the input data or the hand gestures are captured by a camera that will be projected to a local workspace and displayed on the monitor. This set up requires the user to be at certain location, which limits the user mobility. Therefore, Alem*, et al.*[65] and Huang*, et al.*[21] a wearable system that allows the user to move freely since the camera is not fixed anymore as illustrate in Figure 4(D). Their systems operate on one to one relationship. Both remote and local sites are allowed to send the gestures, but only the local site can view the projected image. Alem and Li [20] then investigated the possibility of combining hand gesture and cursor pointer together for video-mediated collaboration. The results revealed that both gestures had almost similar performance since all participants performed the tasks well with only minor mistakes. However, most of the participants preferred a hand gesture pointer instead of the cursor pointer because a gesture is more flexible and can represent more symbols. On the other hand, a pointer can only represent a deictic point and can be misunderstood easily by the other parties.

Current hand pointer applications are still in early stage for teleconsultation usage due to high requirement of the telecommunication bandwidth to convey the images. Hence, it requires an expensive installation cost, which is not feasible for rural area implementation. Moreover, a hand pointer system also requires a more accurate algorithm to perform well such complex modeling of the scene. Paper by Argyros and Lourakis [79] proved that the preprocessing steps required is very challenging such as detection, tracking and recognizing the hand gestures. Gallo and Ciampi [80] improved the system robustness by using a hand glove for more recognizable gestures. However, usage of a glove is unpractical for a telemedicine application due to heavy and bulky size of the glove that hinders the doctor ability to write the diagnosis report at the same time. Besides, the high-tech glove contains a lot of electronic components that require careful handling. The user needs to be trained on how to operate the devices optimally for a safety reason.

However, the usage of a hand pointer system in telemedicine is encouraging, especially for human-computer interface (HCI) applications. An example of hand pointer implementation in telemedicine is to perform sterility procedures in order to avoid bacteria and virus contamination. Hand pointer is manipulated to direct the inexperienced doctor to perform the procedures as shown by Grätzel*, et al.*[81], Grange*, et al.*[82] and Wachs*, et al.*[83]. The gesture assisted system works as follows 1) “push-to-click” is represented by hand pressing movement 2) “wait-to-click” is inferred from the absence of hand movement for a particular length of time. 3) “turn left” command is performed by moving the hand towards left direction. In [84], the authors improved the hand pointer system for dynamic environment usage by considering both hand motion and posture simultaneously for realistic gesture representations. A hand pointer system is also used to zoom in or out the camera perspective remotely by manipulating all five fingers movements [85]. According to Wachs*, et al.*[83], this technology managed to improve surgeon performance by reducing unnecessary movements while discussing with his assistant and browsing through the patient data. Therefore, we strongly believed that a hand pointer system will be further developed given such a vast potential application. Table 1 shows the comparison of each pointer technology.

**Table 1:** Comparison between pointer technologies in telemedicine

**Telepointer**	**Pointer manipulation/ Relationship**	**Hardware requirements**	**Advantage**	**Disadvantage**
Laser	One to many	•Mounted laser pointer	•Expert can point directly to the specific point on the patient	•Not sufficient enough to lead the direction
		•Video camera		
		•Computer devices		
			•Save the search time with more accurate location	
		•Video display		
			•Easily incorporated into the modern operating theatre	
			•Low cost installation.	
Cursor	One to one	•Computer devices	•Easily incorporated into the modern operating theatre since computer is important devices at hospital for management and etc.	•Small graphical pointer on the screen display
	One to many	•Videoconferencing devices		
				•Movement may not be noticeable especially in large screen
				•Doctor at local side needs to look repeatedly at the projected image to see the exact location of the expert’s pointer
			•Low cost installation.	
				•Having to learn how to operate the mouse
				•Insufficient for an effective collaboration
Sketching	One to many	•Portable computer devices (PDA / Tablet)	•Freedom to draw anything without any restriction	•Doctor at local side needs to look repeatedly at the projected image to see the exact location of the expert’s pointer
		•Videoconferencing devices	•Multiple shape and size of pointer increase viewer awareness	
		•Telestrator devices		
		•Stylus pen		
Hands	One to one	•Computer devices	•Can form many shapes of hand gestures	•High cost for installation
		•Face video camera		
				•High bandwidth
				•Complex algorithm
		•Overhead video camera		
		•Screen video camera		
		•Wearable devices		

### Issues and challenges

In the previous subsections, we have discussed the main role and current technology available for the telepointer system. Although it has the ability to increase the quality of health care, issues such as clinician’s concentration, insufficient gesture information and real-life applications should also be addressed.

### Clinician’s concentration

The usage of a cursor pointer on the projector screen or display device is a common practice in medical applications. Because of this practice, a clinician needs to divide their focus between looking at the pointer on the display device and the patient. A clinician needs to look repeatedly at the projected image to see the exact location of the expert’s pointer. Difficulty arises when the expert asks the clinician to search for a specific point on the patient body. This may cause a serious problem, especially during a telesurgery where the focus of attention should be on the patient instead of the display device. Therefore, a telepointer system which is capable of transferring digital gestures to the physical workspace is more suitable for this type of telemedicine application.

### Insufficient gesture information

Laser pointer has the ability to point directly to the specific point on the patient. Thus, it will reduce the search time and a more accurate localization can be obtained. Furthermore, pattern of the laser spot movement can also represent some form of instruction. However, a clinician claimed that normal telepointer without any gesture information is not sufficient enough to lead the direction in complex teleconsultation process [86]. Moreover, laser pointer movement depends only on the cursor movement by the expert who might lead to an error, especially while clicking the interface or taking a glance at the patient’s body. This error will lead to an inaccurate area of interest.

### Real-life applications

A telemedicine system requires more accurate and precise technology since it deals with living things. Most paper [43,45] only considered static patients. However, this assumption does not represent precise real-life applications since human does move a lot. Certain parts of the body such as mouth and eyes move unintentionally as reported by Jon and Bowden [87]. In surgery, internal organs also move unconsciously such as heart pumping, colons contraction and lungs expanding motion. Real-life applications must consider the ability of the pointer to track and point to the accurate spot on the object at a local site. It should reflect the same point as pointed out by the expert at the remote site regardless of the object movement at a local site. Salient features are needed to track the moving components. Tracking process will be more complicated due to poor texture on the human skin and tissue, which will degrade the performance of the salient feature’s detection.

### Conclusions & future works

In this paper, we have discussed state of the art of telepointer technology in telemedicine. Based on recent publications, we can infer that telepointer system is a very valuable support tool for telemedicine applications that improves communication quality between 1) clinicians and other clinicians, 2) clinicians and patients, and 3) medical students and expert clinicians. Telepointer system is a nonverbal communication mode that enhanced the communication capability of the remote user so that more accurate information will be delivered. There are many types of telepointer hardware such as cursor, laser, hand and sketching tool where every tool has their own unique advantages and disadvantages. In addition, we have identified the environments and surroundings where the tools will perform the best. For the teleconsultation purpose, cursor pointer and sketching were usually used as the support tool by the specialists to convey the instruction to the junior clinicians. On the other hand, laser pointer is used to enhance the communication between clinicians and patients.

Currently, several works in CSCW field [23,24] have focused on robustness improvement of the system to unstable network. We also found out that there is a limited study in telepointer performance with respect to the localization, actual patients and outdated instruments, which result in the limited report on real object implementations.

As for future improvement, we believe there are two areas that will enhance the current system significantly. Firstly, the system can be implemented in a parallel processor instead of sequential approach for faster processing speed. Most of the existing telepointer hardwares utilize central processing unit (CPU) as the main processor, which performs moderately slow if the image resolution is high. High performance processor is required for real-time applications, especially in telesurgery where the decision must be made instantly. Therefore, a graphics processing unit (GPU) is a good alternative to CPU for real-time telemedicine system. GPU consists of hundreds of smaller core, while current CPU technology typically has four to eight cores. This feature makes GPU as a powerful computing tool for medical image information. Besides, image resolution and interpolation can be executed at a faster speed. Due to bandwidth limitation, most of the captured images need to be transmitted at a low resolution. Jie *et al.*[88] implemented their down sample method in GPU so that the captured images can be transmitted at a lower resolution. Then, the down sample can be enhanced through image sharpening, which also requires heavy computational load. As a result, their method can perform five times faster than the conventional compression method. By having greater computational ability, hybrid telepointer with high resolution will be utilized more in the telemedicine field. One obvious advantage of using higher-resolution image is better localization of the pointer information such that more accurate pointing can be obtained.

Secondly, a telepointer system can be improved by using a more robust algorithm, especially for the image-processing part. A statistical approach will result in better learning compared to heuristic approach [89]. It will reduce the bottleneck effect when the decision rules are out of the input set such as during illumination changes. Statistical approaches are based on a collection of quantitative data manipulation and interpretation on its statistical properties to discover the pattern, relationship and distribution of the data. Most of the medical images contain nonrigid object and it is hard to model it by using fixed modelling. Statistical method offers the ability to learn from a bundle of data and come out with more robust formulation. This approach has been used in many high level applications [90] and has been applied in various medical systems [91,92]. By using this approach, a better pointer tracking system will be developed to keep track the movement of the pointer device at the remote site. At the same time, the local site pointer can be tuned to follow the remote site movement in order to obtain more accurate feedback data.

## Abbreviations

DOVE: Drawing over video environment; HMD: Head mounted display; PC: Personal computer; CSCW: Computer supported co-operative work; VEDH: Severe vertex epidural hematoma; JPEG: Joint photographic experts group; ECG: Electrocardiogram; MMS: Multimedia Messaging Service; SMS: Short message service; WACL: Wearable active camera/laser; IVCST: Interactive visual content sharing and telestration; PDA: Personal digital assistant; HCI: Human-computer interface; CPU: Central processing unit; GPU: Graphics processing unit; WinVicos: Wavelet-based interactive video communication system; REPETE: REmote patient education in a telemedicine environment architecture.

## Competing interests

The authors declare that they have no competing interest.

## Authors’ contributions

RAK: provision of study material with NZ, analysed literature review and drafted manuscript writing, MAZ: Supervised and revising the manuscript critically, MMM: Supervised and proposed the main idea with IS and NHML. All authors read and approved the final manuscript.

## References

[B1] Latifi R Telepresence and telemedicine in trauma and emergency Studies in health technology and informatics 2008 Amsterdam: Oxford: Ios Press viii, 287 18305337

[B2] Murthy VK Krishnamurthy EV Multimedia Computing Environment for Telemedical Applications Encyclopedia of Information Science and Technology 2005 IGI Global 2045 2050

[B3] Greenberg S Gutwin C Roseman M Semantic telepointers for groupware Computer-human interaction, 1996 proceedings, sixth australian conference on; 24–27 Nov 1996 1996 54 61

[B4] Fussell SR Setlock LD Yang J Ou J Mauer E Kramer ADI Gestures over video streams to support remote collaboration on physical tasks Hum Comput Interact 2004 19 273 309 10.1207/s15327051hci1903_3

[B5] Kuzuoka H Kosaka J Yamazaki K Suga Y Yamazaki A Luff P Heath C Mediating dual ecologies Proceedings of the 2004 ACM conference on Computer supported cooperative work (CSCW '04) 2004 New York, NY, USA: ACM 477 486

[B6] Ou J Fussell SR Chen X Setlock LD Yang J Gestural communication over video stream: supporting multimodal interaction for remote collaborative physical tasks Proceedings of the 5th international conference on Multimodal interfaces(ICMI '03) 2003 New York, NY, USA: ACM 242 249

[B7] Kirk D Fraser DS Rebecca G, Thomas R, Paul A, Ed C, Robin J, Gary O Comparing remote gesture technologies for supporting collaborative physical tasks Proceedings of the SIGCHI Conference on Human Factors in Computing Systems (CHI '06) 2006 New York, NY, USA: ACM 1191 1200

[B8] Tajimi K Sakata N Uemura K Nishida S Remote collaboration using real-world projection interface Systems Man and cybernetics (SMC), 2010 IEEE international conference on; 10–13 Oct. 2010 2010 IEEE 3008 3013

[B9] Ereso AQ Garcia P Tseng E Gauger G Kim H Dua MM Victorino GP Guy TS Live transference of surgical subspecialty skills using telerobotic proctoring to remote general surgeons J Am Coll Surg 2010 211 400 411 10.1016/j.jamcollsurg.2010.05.014 20800198

[B10] Pichiliani MC Hirata CM Soares FS Forster CHQ Bertino E, Joshi JBD TeleEye: an awareness widget for providing the focus of attention in collaborative editing systems collaborative computing: networking, applications and worksharing 2009 10 Heidelberg: Springer Berlin 258 270 [Akan O, Bellavista P, Cao J, Dressler F, Ferrari D, Gerla M, Kobayashi H, Palazzo S, Sahni S, Shen X*, et al.* (Series Editor): *Lecture Notes of the Institute for Computer Sciences, Social Informatics and Telecommunications Engineering*] 10.1007/978-3-642-03354-4_20

[B11] Ikeda S Asghar Z Hyry J Pulli P Pitkanen A Kato H Remote assistance using visual prompts for demented elderly in cooking Proceedings of the 4th International Symposium on Applied Sciences in Biomedical and Communication Technologies (ISABEL '11) 2011 New York, NY, USA: ACM 1 5

[B12] Kurata T Sakata N Kourogi M Kuzuoka H Billinghurst M Remote collaboration using a shoulder-worn active camera/laser Wearable computers, 2004 ISWC 2004 eighth international symposium on; 31 Oct.-3 Nov 2004 IEEE 62 69

[B13] Alem L Tecchia F Huang W Remote tele-assistance system for maintenance operators in mines Proceedings of 11th Underground Coal Operators Conference (COAL2011) 2011 Wollongong University

[B14] Lai AM Kaufman DR Starren J Shea S Evaluation of a remote training approach for teaching seniors to use a telehealth system Int J Med Inform 2009 78 732 744 10.1016/j.ijmedinf.2009.06.005 19620023 PMC2757535

[B15] Christophe B amp, vart Antoine B Hassan Abdourahman A Paul M Renaud D Severe Vertex Epidural Hematoma in a Child: A Case Report of a Management without Expert Neurosurgical Care Case Reports in Surgery 2011 10.1155/2011/476416 PMC3350272 22606578

[B16] McLauchlan CA Jones K Guly HR Interpretation of trauma radiographs by junior doctors in accident and emergency departments: a cause for concern? J Accid Emerg Med 1997 14 295 298 10.1136/emj.14.5.295 9315930 PMC1343093

[B17] Wootton R Telemedicine support for the developing world J Telemed Telecare 2008 14 109 10.1258/jtt.2008.003001 18430271

[B18] Azzolini C A pilot teleconsultation network for retinal diseases in ophthalmology J Telemed Telecare 2011 17 20 24 10.1258/jtt.2010.100305 21097561

[B19] Beula Devamalar PM Thulasi Bai V Srivatsa SK Design and architecture of real time web-centric tele health diabetes diagnosis expert system Int J Med Eng and Informatics 2009 1 307 317 10.1504/IJMEI.2009.022642

[B20] Alem L Li J A study of gestures in a video-mediated collaborative assembly task Adv in Hum-Comp Int 2011 2011 1 7

[B21] Huang W Alem L Albasri J HandsInAir: a wearable system for remote collaboration CoRR 2011 abs/1112.1742

[B22] Sachpazidis I Kiefer S Selby P Ohl R Sakas G A medical network for teleconsultations in Brazil and Colombia 2006 16 21

[B23] Pavlovych A Stuerzlinger W Target following performance in the presence of latency, jitter, and signal dropouts Canadian human-computer communications society 2011 33 40

[B24] Dyck J Gutwin C Subramanian S Fedak C High-performance telepointers Proceedings of the 2004 ACM conference on Computer supported cooperative work (CSCW '04) 2004 New York, NY, USA: ACM 172 181

[B25] Gutwin C Benford S Dyck J Fraser M Vaghi I Greenhalgh C Revealing delay in collaborative environments Proceedings of the SIGCHI Conference on Human Factors in Computing Systems (CHI '04) 2004 New York, NY, USA: ACM 503 510

[B26] Rizou D Sesma L Salvatore L Doctors mobility covered by TraumaStation. In information technology and applications in biomedicine (ITAB) 2010 10th IEEE International Conference on; 3–5 Nov 2010 2010 1 421937301

[B27] Telemedicinehttp://www.ustudy.in/node/5162

[B28] Bonnardot L Rainis R Store-and-forward telemedicine for doctors working in remote areas J Telemed Telecare 2009 15 1 6 10.1258/jtt.2008.008004 19139214

[B29] Dala-Ali BM Lloyd MA Al-Abed Y The uses of the iPhone for surgeons Surgeon 2011 9 44 48 10.1016/j.surge.2010.07.014 21195331

[B30] Farber N Haik J Liran A Weissman O Winkler E Third generation cellular multimedia teleconsultations in plastic surgery J Telemed Telecare 2011 17 199 202 10.1258/jtt.2010.100604 21508079

[B31] Hailey D Ohinmaa A Roine R Study quality and evidence of benefit in recent assessments of telemedicine J Telemed Telecare 2004 10 318 324 10.1258/1357633042602053 15603628

[B32] Augestad K Lindsetmo R Overcoming distance: video-conferencing as a clinical and educational tool among surgeons World J Surg 2009 33 1356 1365 10.1007/s00268-009-0036-0 19384459 PMC2691934

[B33] Lundvoll Nilsen L Collaboration and learning in medical teams by using video conference Behav & Inf Technol 2011 30 507 515 10.1080/0144929X.2011.577193

[B34] Nagata H Mizushima H A remote collaboration system for telemedicine using the Internet J Telemed Telecare 1998 4 89 94 10.1258/1357633981932019 9744164

[B35] Young-Hughes S Simbartl L Spinal cord injury/disorder teleconsultation outcome study Rehabil Nurs 2011 36 153 10.1002/j.2048-7940.2011.tb00083.x 21721396

[B36] Butner SE Ghodoussi M Transforming a surgical robot for human telesurgery Robotics and Automation, IEEE Transactions on 2003 19 818 824 10.1109/TRA.2003.817214

[B37] Clifford G Blaya J Hall-Clifford R Fraser H Medical information systems: a foundation for healthcare technologies in developing countries Biomed Eng Online 2008 7 18 10.1186/1475-925X-7-18 18547411 PMC2447839

[B38] Ramalanjaona G The development of telemedicine in Madagascar J Telemed Telecare 2011 17 161 162 10.1258/jtt.2010.101003 21320921

[B39] Xia S Sun D Chengzheng S Chen D Object-associated telepointer for real-time collaborative document editing systems Collaborative computing: networking, applications and worksharing, 2005 international conference on; 0–0 0 2005 10

[B40] Sun A Sun C Televiewpointer: an integrated workspace awareness widget for real-time collaborative 3d design systems Proceedings of the 16th ACM international conference on Supporting group work (GROUP '10) 2010 New York, NY, USA: ACM 21 30

[B41] Julsrud PR Breen JF Jedeikin R Peoples W Wondrow MA Bailey KR Telemedicine consultations in congenital heart disease: assessment of advanced technical capabilities Mayo Clin Proc 1999 74 758 763 10.4065/74.8.758 10473350

[B42] Kaidu M Toyabe S Oda J Okamoto K Ozaki T Shiina M Sasai K Akazawa K Development and evaluation of a teleradiology and videoconferencing system J Telemed Telecare 2004 10 214 218 10.1258/1357633041424430 15273031

[B43] Lee JS Tsai CT Pen CH Lu HC A real time collaboration system for teleradiology consultation Int J Med Inform 2003 72 73 79 10.1016/S1386-5056(03)00130-8 14644308

[B44] Gambadauro P Magos A NEST (network enhanced surgical training): A PC-based system for telementoring in gynaecological surgery Eur J Obstet Gynecol Reprod Biol 2008 139 222 225 10.1016/j.ejogrb.2007.12.004 18243486

[B45] Ohta S Kuzuoka H Noda M Sasaki H Mishima S Fujikawa T Yukioka T Remote support for emergency medicine using a remote-control laser pointer J Telemed Telecare 2006 12 44 48 10.1258/135763306775321362 16438779

[B46] Bruschi M Micali S Porpiglia F Celia A De Stefani S Grande M Scarpa RM Bianchi G Laparoscopic telementored adrenalectomy: the italian experience Surg Endosc 2005 19 836 840 10.1007/s00464-004-9124-2 15880286

[B47] Yuan-Pin Y Object oriented teleconsultations in global PACS using multi-thread Java System Sciences, 1997, Proceedings of the Thirtieth Hawaii International Conference on; 7-10 Jan 1997 1997 IEEE 166 175 vol.164

[B48] Bardram JE Activity-based computing for medical work in hospitals ACM Trans Comput-Hum Interact 2009 16 1 3622563232

[B49] Graschew G Roelofs TA Rakowsky S Schlag PM WinVicos - wavelet-based interactive video communication system for medical and non-medical applications Industrial Technology, 2002 IEEE ICIT '02 2002 IEEE International Conference on; 11-14 Dec. 2002 2002 IEEE 864 868 vol.862

[B50] Gackowski A Czekierda Ł Chrustowicz A Cała J Nowak M Sadowski J Podolec P Pasowicz M Zieliński K Development, implementation, and multicenter clinical validation of the TeleDICOM—advanced, interactive teleconsultation system J Digit Imaging 2011 24 541 551 10.1007/s10278-010-9303-8 20495992 PMC3092051

[B51] Lai AM Starren JB Kaufman DR Mendonça EA Palmas W Nieh J Shea S The REmote patient education in a telemedicine environment architecture (REPETE) Telemed E-Health 2008 14 355 361 10.1089/tmj.2007.0066 18570565

[B52] MARSHALL J The safety of laser pointers: myths and realities Br J Ophthalmol 1998 82 1335 1338 10.1136/bjo.82.11.1335 9924345 PMC1722403

[B53] Silva A Frere A Virtual environment to quantify the influence of colour stimuli on the performance of tasks requiring attention Biomed Eng Online 2011 10 74 10.1186/1475-925X-10-74 21854630 PMC3201025

[B54] Schneider A Wilhelm D Bohn U Wichert A Feussner H An evaluation of a surgical telepresence system for an intrahospital local area network J Telemed Telecare 2005 11 408 413 10.1258/135763305775013518 16356315

[B55] Kirstein C Muller H Interaction with a projection screen using a camera-tracked laser pointer Multimedia Modeling, 1998 MMM '98 Proceedings 1998; 12-15 Oct 1998 1998 IEEE 191 192

[B56] Dan R Olsen J Nielsen T Laser pointer interaction Proceedings of the SIGCHI conference on Human factors in computing systems; Seattle, Washington, United States 2001 New York, NY, USA: ACM 17 22

[B57] Ahlborn BA Thompson D Kreylos O Hamann B Staadt OG A practical system for laser pointer interaction on large displays Proceedings of the ACM symposium on Virtual reality software and technology; Monterey, CA, USA 2005 New York, NY, USA: 106 109

[B58] Ursic CM Coates NE Fischer RP The pocket laser pointer as a teaching tool in laparoscopic surgery Surg Laparosc Endosc Percutan Tech 1997 7 47 48 10.1097/00019509-199702000-00012 9116947

[B59] Racz GB Kao JY Laser pointer as a teaching tool in operating rooms Anesthesiology 1998 88 549 9477086 10.1097/00000542-199802000-00044

[B60] Moustris GP Hiridis SC Deliparaschos KM Konstantinidis KM Evolution of autonomous and semi-autonomous robotic surgical systems: a review of the literature Int J Med Robot 2011 7 375 392 10.1002/rcs.408 21815238

[B61] Clarke JV Deakin AH Nicol AC Picard F Measuring the positional accuracy of computer assisted surgical tracking systems Comput Aided Surg 2010 15 13 18 10.3109/10929081003775774 20433317

[B62] Yamazaki K Yamazaki A Kuzuoka H Oyama S Kato H Suzuki H Miki H Bødker S, Kyng M, Schmidt K GestureLaser and GestureLaser Car ECSCW ’99 2002 Netherlands: Springer 239 258

[B63] Ko R Razvi H C-Arm laser positioning device to facilitate percutaneous renal access Urology 2007 70 360 361 10.1016/j.urology.2007.05.013 17826509

[B64] Yamashita N Kaji K Kuzuoka H Hirata K Improving visibility of remote gestures in distributed tabletop collaboration Proceedings of the ACM 2011 conference on Computer supported cooperative work 2011 Hangzhou, China: ACM 95 104

[B65] Alem L Tecchia F Huang W Alem L, Huang W HandsOnVideo: towards a gesture based mobile AR system for remote collaboration recent trends of mobile collaborative augmented reality systems 2011 New York: Springer 135 148

[B66] Tang JC Minneman SL Videodraw: a video interface for collaborative drawing ACM Trans Inf Syst 1991 9 170 184 10.1145/123078.128729

[B67] Anvari M Telesurgery: remote knowledge translation in clinical surgery World J Surg 2007 31 1545 1550 10.1007/s00268-007-9076-5 17534550

[B68] Tsuda S Tichansky MDFDS, Morton MDMPHJ, Jones DB Teleproctoring in surgery the SAGES manual of quality, outcomes and patient safety 2012 US: Springer 513 517

[B69] Ali MR Loggins JP Fuller WD Miller BE Hasser CJ Yellowlees P Vidovszky TJ Rasmussen JJ Pierce J 3-D telestration: a teaching tool for robotic surgery J Laparoendosc Adv Surg Tech 2008 18 107 112 10.1089/lap.2007.0051 18266586

[B70] Qiru Z Dong L Interactive visual content sharing and telestration: a novel network multimedia service Intelligence in next generation networks (ICIN), 2010 14th international conference on; 11–14 Oct. 2010 2010 1 6

[B71] Kim H Cho Y Do E Jacko J Using Pen-based computing in technology for health human-computer interaction. Users and applications Lecture notes in computer science 2011 Berlin / Heidelberg: Springer 192 201 6764

[B72] Dante A-T The design and evaluation of a Pen-based computer interface for novice older users Computer science, 2006 ENC ’06 seventh mexican international conference on; sept. 2006 2006 142 150

[B73] Westin J Ghiamati S Memedi M Nyholm D Johansson A Dougherty M Groth T A new computer method for assessing drawing impairment in Parkinson’s disease J Neurosci Methods 2010 190 143 148 10.1016/j.jneumeth.2010.04.027 20438759

[B74] Kim H Exploring technological opportunities for cognitive impairment screening Book exploring technological opportunities for cognitive impairment screening (editor ed.^eds.). pp. 887–892 2011 ACM 887 892

[B75] Tu H Ren X Zhai S A comparative evaluation of finger and pen stroke gestures Book a comparative evaluation of finger and pen stroke gestures (editor ed.^eds.). pp. 1287–1296 2012 ACM 1287 1296

[B76] Yun F Huang TS HMouse: head tracking driven virtual computer mouse Applications of computer vision, 2007 WACV ’07 IEEE workshop on; Feb. 2007 2007 30 30

[B77] Olsen A Schmidt A Marshall P Sundstedt V Using eye tracking for interaction Book using eye tracking for interaction (editor ed.^eds.). pp. 741–744 2011 ACM 741 744

[B78] Wickey A Alem L Analysis of hand gestures in remote collaboration: some design recommendations Proceedings of the 19th Australasian conference on Computer-Human Interaction: Entertaining User Interfaces; Adelaide, Australia 2007 New York, NY, USA: ACM 87 93

[B79] Argyros A Lourakis MA Huang T Sebe N Lew M Pavlović V Kölsch M Galata A Kisačanin B Vision-Based Interpretation of Hand Gestures for Remote Control of a Computer Mouse Computer Vision in Human-Computer Interaction. Volume 3979 Huang T, Sebe N, Lew M, Pavlović V, Kölsch M, Galata A, Kisačanin B 2006 Springer Berlin Heidelberg 40 51 Lecture Notes in Computer Science

[B80] Gallo L Ciampi M Wii remote-enhanced hand-computer interaction for 3D medical image analysis Current trends in information technology (CTIT), 2009 international conference on the; 15–16 Dec. 2009 2009 1 6

[B81] Grätzel C Fong T Grange S Baur C A non-contact mouse for surgeon-computer interaction Technol Health Care 2004 12 245 257 15328453

[B82] Grange S Fong T Baur C ACM, New York, NY, USA M/ORIS: a medical/operating room interaction system Proceedings of the 6th international conference on Multimodal interfaces 2004 New York N, USA: State College, PA, USA 159 166

[B83] Wachs JP Stern HI Edan Y Gillam M Handler J Feied C Smith M A gesture-based tool for sterile browsing of radiology images J Am Med Inform Assoc 2008 15 321 323 18451034 10.1197/jamia.M241 PMC2410001

[B84] Wachs J Stern H Edan Y Gillam M Feied C Smith M Handler J Saad A, Dahal K, Sarfraz M, Roy R, Saad A, Dahal K, Sarfraz M, Roy R Gestix: A Doctor-Computer Sterile Gesture Interface for Dynamic Environments Soft Computing in Industrial Applications. Volume 39 2007 Springer Berlin Heidelberg 30 39 Advances in Soft Computing

[B85] MacLean J Herpers R Pantofaru C Wood L Derpanis K Topalovic D Tsotsos J Fast hand gesture recognition for real-time teleconferencing applications Recognition, Analysis, and Tracking of Faces and Gestures in Real-Time Systems, 2001 Proceedings IEEE ICCV Workshop on 2001 IEEE 133 140

[B86] Jayaraman S Apriasz I Trejos AL Bassan H Patel RV Schlachta CM Novel hands-free pointer improves instruction efficiency in laparoscopic surgery Surg Innov 2009 16 73 77 19117977 10.1177/1553350608329802

[B87] Eng-Jon O Bowden R Robust facial feature tracking using shape-constrained multiresolution-selected linear predictors Pattern Analysis and Machine Intelligence, IEEE Transactions on 2011 33 1844 1859 10.1109/TPAMI.2010.205 21135441

[B88] Jie C Ming-Chao C Xiaolin W Jie L GPU-aided directional image/video interpolation for real time resolution upconversion Multimedia Signal Processing, 2009 MMSP '09 IEEE International Workshop on 2009 IEEE 1 6

[B89] Zulkifley M Moran B Maino G, Foresti G Statistical patch-based observation for single object tracking image analysis and processing – ICIAP 2011 Lecture notes in computer science 2011 Heidelberg: Springer Berlin 119 129 6979

[B90] Zulkifley MA Moran B Rawlinson D Robust foreground detection: a fusion of masked GreyWorld, probabilistic gradient information and extended conditional random field approach Sensors-Basel 2012 12 5623 5649 10.3390/s120505623 22778605 PMC3386704

[B91] Vidhya M Statistical analysis identifies genes in prostate cancer Int J Medical Eng and Inf 2009 1 407 417

[B92] Xue Z Shen D A new statistically-constrained deformable registration framework for MR brain images Int J Medical Eng and Inf 2009 1 357 367 10.1504/IJMEI.2009.022646 PMC2792917 20011232

